# Rev-erbα exacerbates hepatic steatosis in alcoholic liver diseases through regulating autophagy

**DOI:** 10.1186/s13578-021-00622-4

**Published:** 2021-07-10

**Authors:** Qingxue Liu, Lei Xu, Meifei Wu, Yiwen Zhou, Junfa Yang, Cheng Huang, Tao Xu, Jun Li, Lei Zhang

**Affiliations:** 1 School of Pharmacy, Anhui Institute of Innovative Drugs, Anhui Medical University, Hefei, 230032 Anhui China; 2 Inflammation and Immune Mediated Diseases Laboratory of Anhui Province, Hefei, 230032 People’s Republic of China; 3 The Key Laboratory of Anti-Inflammatory and Immune Medicines, Ministry of Education, Hefei, 230032 China

**Keywords:** Rev-erbα, AFL, Autophagy, Bmal1, Lysosome

## Abstract

**Background and aims:**

Alcoholic fatty liver (AFL) is a liver disease caused by long-term excessive drinking and is characterized by hepatic steatosis. Understanding the regulatory mechanism of steatosis is essential for the treatment of AFL. Rev-erbα is a member of the Rev-erbs family of nuclear receptors, playing an important role in regulating lipid metabolism. However, its functional role in AFL and its underlying mechanism remains unclear.

**Results:**

Rev-erbα was upregulated in the liver of EtOH-fed mice and EtOH-treated L-02 cells. Further, Rev-erbα activation exacerbates steatosis in L-02 cells. Inhibition/downexpression of Rev-erbα improved steatosis. Mechanistically, autophagy activity was inhibited in vivo and vitro. Interestingly, inhibition/downexpression of Rev-erbα enhanced autophagy. Furthermore, silencing of Rev-erbα up-regulated the nuclear expression of Bmal1. Autophagy activity was inhibited and steatosis was deteriorated after EtOH-treated L-02 cells were cotransfected with Rev-erbα shRNA and Bmal1 siRNA.

**Conclusions:**

Rev-erbα induces liver steatosis, which promotes the progression of AFL. Our study reveals a novel steatosis regulatory mechanism in AFL and suggest that Rev-erbα might be a potential therapeutic target for AFL.

**Supplementary Information:**

The online version contains supplementary material available at 10.1186/s13578-021-00622-4.

## Introduction

Chronic alcohol consumption is a crucial factor contributing to alcoholic liver disease (ALD). ALD includes a broad spectrum of liver disorders, ranging from alcoholic fatty liver (AFL), alcoholic steatohepatitis (ASH), alcoholic fibrosis (AF), alcoholic cirrhosis (AC) to alcoholic hepatocellular carcinoma (AHCC) [1–3]. Alcoholic fatty liver disease (AFLD) has become a global healthcare problem. Among AFLD, AFL is the earliest phase characterized by ballooning of hepatocytes, lipid droplets deposition and inflammatory cells infiltration. Hepatic steatosis is a progression of excessive triglyceride accumulation caused by the imbalance between lipids synthesis and oxidation [4]. As the main pathological factor, lipid accumulation plays a pivotal role in the occurrence and progression of AFL [5, 6], although mild fatty liver can be alleviated by exercise and diet. However, the regulatory mechanism of steatosis remains to be supplemented.

Rev-erbα is an orphan nuclear receptor and belongs to the nuclear receptor family. Accumulating studies suggest that Rev-erbα is associated with many diseases, including hyperlipidemia, hyperglycemia and liver diseases such as liver fibrosis and cancer [7–9]. Among this, Rev-erbα is closely related to lipid metabolism, which is supported by the fact that Rev-erbα can promote the differentiation of 3T3-L1 preadipocytes and enhance lipid storage [10]. Moreover, Rev-erbα regulates lipoproteinase and triglyceride by directly inhibiting the activity of ApoC-III [11, 12]. As a transcriptional repressor, Rev-erbα has a dual role in regulating lipid metabolism. Fontaine et al. pointed out that lipid deposition increased significantly in mice lacking Rev-erbα [13]. Consistent with this view, Sitaula et al. found that Rev-erbα could repress transcription of cholesterol biosynthesis genes by promoting recruitment of NCoR and HDAC3, resulting in reducing cholesterol levels and biosynthesis in mice [14]. However, the function of Rev-erbα in the pathogenesis of AFL and whether Rev-erbα regulates hepatic steatosis remains unclear.

Autophagy is a protective mechanism that removes lipid droplets, protein aggregates, and damaged organelles from hepatocytes [15]. Growing evidence highlights the involvement of autophagy in regulating hepatic lipid metabolism. It has been reported that inhibition of autophagy in hepatocyte could increase the storage of triglyceride in lipid conjoined as well as inhibit the degradation of lipid droplets [16]. Moreover, lack of autophagy activity alters fatty liver and liver injury condition caused by alcohol [17]. Attractively, it was pointed out that Rev-erbα can represses autophagosome formation and lysosomal biogenesis directly in skeletal muscle [18, 19]. Grimaldi et al. reported that Rev-erbα can inhibit the formation of autophagy, which blocked the source of cancer nutrition [20]. More importantly, autophagy genes in zebrafish is under the control of Rev-erbα [21]. Given the critical role of autophagy on lipid metabolism and the intimate relationship between Rev-erbα and autophagy, we aimed to investigate whether Rev-erbα promotes lipid accumulation in AFL by affecting autophagy activity.

In this study, we found that Rev-erbα was significantly increased both in vitro and in vivo. Following inhibition of Rev-erbα, hepatic steatosis was ameliorated with the improvement of autophagy activity. Mechanistic studies suggest that Rev-erbα inhibited autophagy by regulating Bmal1expression. Taken together, our results elucidated that Rev-erbα accelerated lipid deposition by inhibiting autophagy. We sought to defined the potential roles of Rev-erbα in hepatic steatosis and the molecular mechanisms underlying this regulation in AFL.

## Materials and methods

### Reagents and antibodies

Primary antibodies Rev-erbα, Bmal1 and Beclin1 were purchased from Proteintech Wu Han China. Primary antibodies LC3 and P62 were purchased from Cell Signaling Technology USA. Primary antibodies Pparα and Srebp1c were purchased from Affinity Biosciences USA. β-Actin, secondary antibodies and goat anti-mouse Alexa Fluor 488 were purchased from Zhongqiao Jinshan China. Nucleoprotein/cytoplasmic protein extraction kit was purchased from Best Bio Shanghai, China. GSK4112 (CAS number: 1216744–19-2) was purchased from TargetMol China. SR8278 (CAS number: 1254944-66-5) was purchased from APExBIO USA. ORO was purchased from Sigma USA. Rev-erbα expression interference plasmid and Control plasmid was purchased from GenePharma (ShangHai, China). Bmal1 small interfering RNA (siRNA) and Control siRNA were synthesized by GenePharma (Shanghai, China).

### Cell culture

L-02 cells, the human normal liver cell line was purchased from Type Culture Collection of the Chinese Academy of Sciences (Shanghai, China), the cell line was cultured with DMEM medium (Hyclone, Salt Lake City, UT, USA) supplemented with 10% fetal bovine serum (FBS; Hangzhou Sijiqing Biological Engineerin materials, China) and 1% Penicillin–Streptomycin (Biyuntian, China). The incubator was maintained at 37 °C and contains 5% CO_2_. The medium was changed once a day. Cells were treated with 150 mM ethanol at a fixed time of the day, at 5 p.m. for 48 h. At least three independent experiments were performed throughout study.

### Animal model of AFL

Male C57BL/6J mice (20–22 g, 6–8 weeks) were purchased from Laboratory Animal Center of Anhui Medical University. All animal experiments were approved by Anhui Medical University Animal Experimental Ethics Committee (Number: LLSC20150348). Animal experiments took place at the Animal Experiment Center in Anhui Medical University. Mice were kept in a Controlled temperature (25 ± 1 °C) and humidity (50 ± 5%) environment with a 12 h light/dark cycle with ad lib access to food and water. Mice were randomly divided into Control diet (CD-fed), ethanol diet (EtOH-fed). Ethanol diet feeding and binge was performed with the protocol described by Gao-Binge [22]. According to protocol, mice were fed the regular Lieber-DeCarli normal diet or ethanol diet (Nantong troffi, CAS number: TP4030) containing increasing (1%–5% vol/vol) ethanol for the adaptation period (5 days) and modeling (10 days) with 5% vol/vol ethanol liquid diet at 5 o'clock every afternoon. Half an hour after feeding, SR8278 group were injected with SR8278 (dissolve in 0.4% DMSO, 99.6% PBS), EtOH-fed group were injected with blank solvent (0.4% DMSO, 99.6% PBS) at 2 mg/kg on Day 13 for 3 days via tail vein. SR8278 group and EtOH-fed mice were gavaged with one time ethanol binge [1.5 mL/100 g, 31.5% (vol/vol)] at 21 p.m. on the last day. Mice were anesthetized by injecting with 1% pentobarbital. Blood and liver tissues of mice were separated for experiments. The mice were euthanized by cervical dislocation. At least six independent experiments were performed in the study.

### Western blotting

Liver tissues and cultured cells were lysed with RIPA lysis buffer (Biyuntian, China), the protein concentration was measured by BCA protein assay kit (Boster, China), the extracted protein samples were separated by 10% or 12% SDS–polyacrylamide gel and then transferred to polyvinyl difluoride membranes (Millipore, USA). After blocking, the membranes were incubated overnight at 4 °C with primary antibody β-actin (1:1000), Rev-erbα (1:1000), Bmal1 (1:1000), LC3 (1:1000), Srebp1c (1:1000), Pparα (1:1000), Beclin1 (1:1000) and P62 (1:1000) for 24 h and then incubated with secondary antibody (1:10,000) for 1 h at room temperature. The membranes were visualized using an ECL-chemiluminescent kit (ECL-plus, Thermo Scientific) and exposed to electrochemiluminescence (GE Healthcare Bio-Sciences, AB, Uppsala, Sweden). The intensities of bands were quantified by using the Image J software (NIH, Bethesda, MD, USA).

### RNA extraction and quantitative real-time PCR

Total RNA was isolated from liver tissue or L-02 cells using the TRIzol reagent (Invitrogen, United States), RNA concentration was measured by Nano Drop 2000 Spectrophotometer (Thermo Scientific, USA). Then the RNA was converted into cDNA by the Reverse transcription kit (TaKaRa, QIAGEN, Japan). Realtime quantitative PCR analyses for mRNA were performed by using cDNA TB-Green real-time PCR Master Mix (TaKaRa, QIAGEN, Japan). β-Actin was used as an internal Control. The sequences of primers used in this article were list in Table 1.

**Table 1:** Primer sequences used in real-time PCR

Genes	Forward primer (5′–3′)	Reverse primer (5′–3′)
(Human) Rev-erbα	TTGAGTCAAGGTCCAGT TTGAATG	GGAGTCCAGGGTCGTC ATGT
(Human) Rev-erbβ	GTCAAGGTCCAGTTTGA ATG	GCGAGATCACCATTCTT GGG
(Human) Pparα	GCACCTGGAGGTATCG TCGAT	CATGGGACCCTTATCA ATCCTAATC
(Human) Srebp1c	GGGTCAGTTGTCCCTTC TCA	TGAGACGTGCCAGACT TCTT
(Human) BMAL1	AGCTGCCTCGTCGCAAT T	CCGTTCACTGGTTGTGG AACT
(Human) β-actin	CCCTGGAGAAGAGCTA CGAG	TGCTAGGAGCCAGAGC AGTA
(Mouse) Rev-erbα	CCCAACGACAACAACC TTTT	CCCTGGCGTAGACCAT TCAG
(Mouse) Rev-erbβ	GGTTAGGTTTGTGAGTG TCCACAGC	GGAAGTGCTCCAACAA GGTAGTGCA
(Mouse) Pparα	AGCTGGTGTAGCAAGT GT	TCTGCTTTCAGTTTTGC TTT
(Mouse) Srebp1c	ACACAGCAACCAGAAA CTCAAG	AGTGTGTCCTCCACCTC AGTCT
(Mouse) β-actin	AGTGTGACGTTGACATC CG	TGCTAGGAGCCAGAGC AGTA

### Immunofluorescence

Cells cultured in 6-well were washed by PBS 3 times and fixed in 4% formaldehyde for 15 min. Then washed by PBS 3 times and blocked by BSA for 30 min at room temperature. Next, cells were washed with PBS 3 times and permeabilized with 1% Triton X-100 solution for 5 min. After washed, cells were incubated with Rev-erbα primary antibody (1:50) overnight at 4 °C. Next day, cells were incubated with secondary antibody at dark for 1 hand counterstained with DAPI for 5 min. At last stained sections were examined by using confocal microscopy (Zeiss, Germany).

### Serum biochemical analysis

The activities of serum alanine aminotransferase (ALT), triglyceride (TG) and total cholesterol (T-CHO) in serum were measured using commercial assay kits (Jiancheng, Nanjing) by microplate reader (Biotek, USA) at appropriate wavelength.

### ORO staining

Cells in the 6-well plates were washed 3 times by PBS, then fixed with 4% Paraformaldehyde for 15 min, After washed 3 times by PBS, sections were stained with working solution ORO (prepared freshly at 25 °C) for 30 min. Finally, sections were washed with 60% dimethylcarbinol and double distilled water. The fat drops were observed by inverted fluorescent microscope. The same operation was introduced to liver tissue after fixing.

### Morphological assessment

Liver tissues were fixed with 4% paraformaldehyde for 24 h, then embedded in paraffin blocks and stained with hematoxylin and eosin (H&E). Immunohistochemistry (IHC) was performed according to a standard procedure. The pathological changes were assessed by a digital pathology slide scanner (3 DHISTECH, Hungary). The IHC results were quantitatively analyzed by the Image-ProPlus Software (MEDIA CYBERNETICS, USA) to calculate the integral optical density (IOD).

### Cell transfection

To down-regulate the expression level of Rev-erbα, L-02 cells were transfected with 1 μg Rev-erbα shRNA (100 μg) by using lipofectamine^TM^2000 (Invitrogen, MA) according to the manufacturer’s instructions. In order to silence Bmal1, Bmal1 siRNA were transfected in L-02 cells by using lipofectamine^TM^2000. After 6 h transfection, the medium was changed to DMEM and ethanol was added. Cells were maintained at 37 °C in a CO_2_ incubator and then collected by trypsin treatment for qRT-PCR and Western blotting analysis. Rev-erbα shRNA sequence: GCTGAATGGCATGGTGT TACT. Bmal1 siRNA sequence: GCACAUCGUGUUAUGAAUATTUAUUCA UAACA CGAUGUGCTT.

### Transmission electron microscope

Liver tissue sample at grain size was fixed in 2.5% glutaraldehyde at 4 °C and placed in 1% osmium tetroxide for 4 h on ice. Next sections were dehydrated in a graded series of ethanol and embedded in LR White resin after washed by 0.1 M sodium cacodylate (pH 7.4). Embedded samples were detected by a transmission electron microscope (Hitachi-7800, Japan).

### Lysosomal acid analysis

Lyso Tracker Green DND-26 was selectively labeled for acidic lysosome in living cells. After cell slides and certain treatment, discard the medium and add 50 nM Lyso Tracker Green DND-26, cells were incubated for 5 min under growth, then switched to fresh medium and detected by laser confocal microscope. The intensity of fluorescence intensity represents the acidity of lysosomes.

### Statistical analysis

All data presented were representative of at least 3 repeat experiments and expressed as mean ± SEM. Statistical analyses were performed with SPSS 17.0 software (Statistical Program for Social Sciences). One-way analysis of variance (ANOVA) was used to evaluate differences between each groups. The differences between two groups were determined by unpaired two-tailed t-test. Results were considered statistically significant with *p* value < 0.05.

## Results

### Rev-erbα is up-regulated in the liver of EtOH-fed mice

**Fig. 1:**
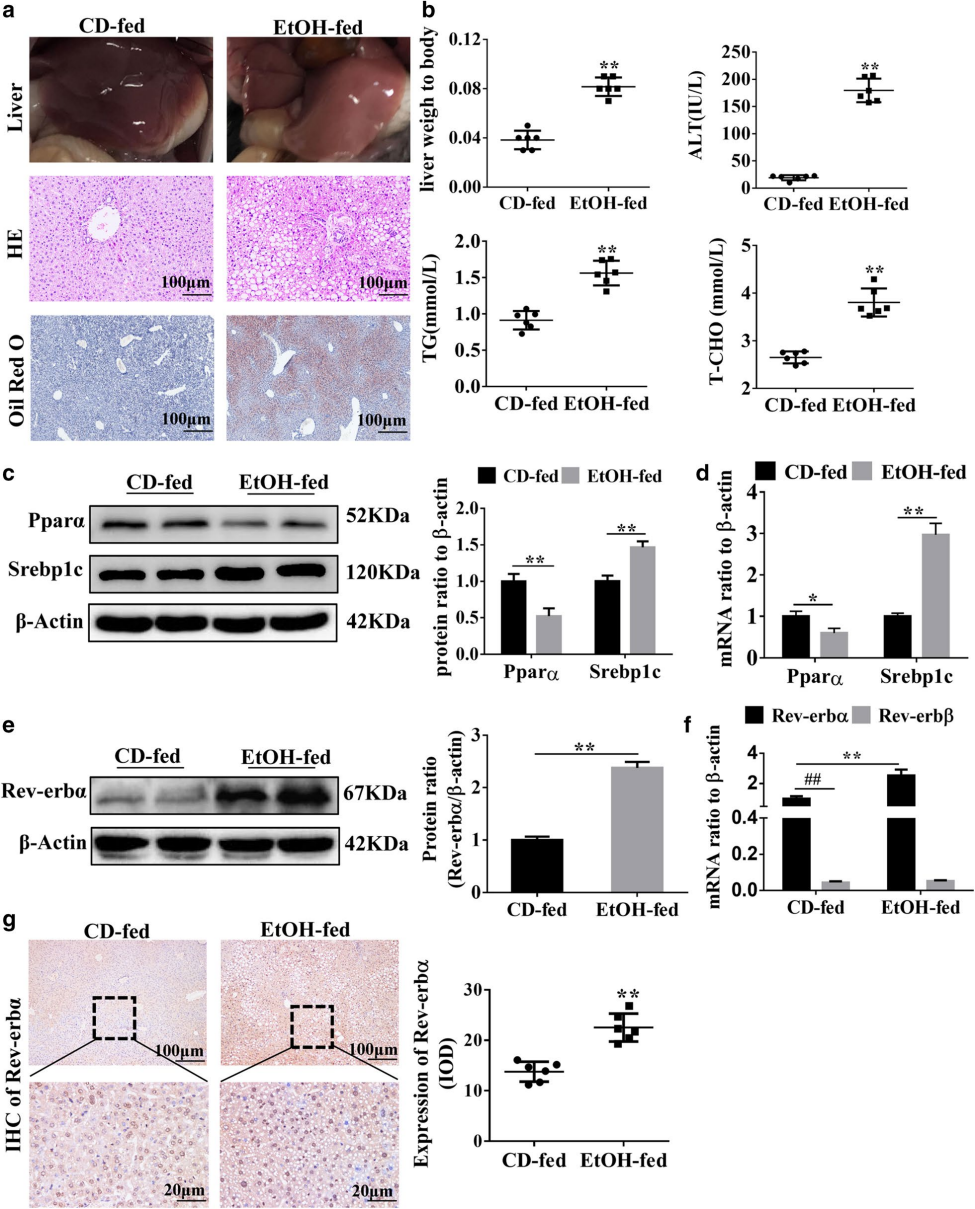
Rev-erbα was up-regulated in the liver of EtOH-fed mice in vivo*.* C57 male mice weigh 20 kg or more were fed EtOH liquid diet or the Control diet for 16 days. **A** Representative image of liver and histological assessment of hepatic pathologic alterations by H&E and ORO staining in alcoholic fatty liver mice (scale bar = 100 μm). **B** The liver weight ratio to body of mice, serum ALT, TG and TC levels in CD-fed mice and EtOH-fed mice. **C**, **D** Western blot and qRT-PCR analysis of Pparα and Srebp1c in CD-fed mice and EtOH-fed mice. **E** Western blot analysis of Rev-erbα in CD-fed mice and EtOH-fed mice. **F** qRT-PCR analysis of Rev-erbα and Rev-erbβ in CD-fed mice and EtOH-fed mice. **G** HIC shows the expression of Rev-erbα in CD-fed mice and EtOH-fed mice (scale bar = 100 μm or 20 μm) (n = 6). Bar represents the mean ± SEM. Significance *P < 0.05, **P < 0.01 vs. CD-fed group. ^#^*P* < 0.05, ^##^*P* < 0.01 vs. Rev-erbα in CD-fed group

We performed chronic EtOH plus single EtOH binge feeding in mice, which is well described by the NIAAA model protocol [22]. As shown in Fig. 1A, compared to CD-fed mice, apparent lipid deposition was showed in the liver of EtOH-fed mice. H&E staining showed that mice in the EtOH-fed group developed steatosis and fat droplets fill the hepatocytes, especially those hepatocytes located around the central vein. ORO staining indicated aggravated steatosis in the EtOH-fed mice compared to control mice. The ratio of liver to body weight, serum ALT, TG and T-CHO levels were all increased in EtOH-fed mice compared to CD-fed mice (Fig. 1B). Results of western blot and qRT-PCR showed that Pparα was decreased and Srebp1c was increased in EtOH-fed mice compared to CD-fed mice (Fig. 1C, D).

To explore whether Rev-erbs was involved in the pathogenesis of AFL, we detected the expression of two subtypes of Rev-erbs. qRT-PCR analysis showed that the level of Rev-erbα was higher than Rev-erbβ in CD-fed group and Rev-erbα was enhanced but Rev-erbβ had no obvious change in EtOH-fed group compared to CD-fed group (Fig. 1F). The higher expression of Rev-erbα in EtOH-fed group was further confirmed by western blot and immunohistochemistry analysis (Fig. 1E, G). These results indicated that it may be Rev-erbα but not Rev-erbβ that plays a critical role in the development of AFL.

### Rev-erbα is up-regulated in EtOH-treated L-02 cells and mediated liver steatosis

**Fig. 2:**
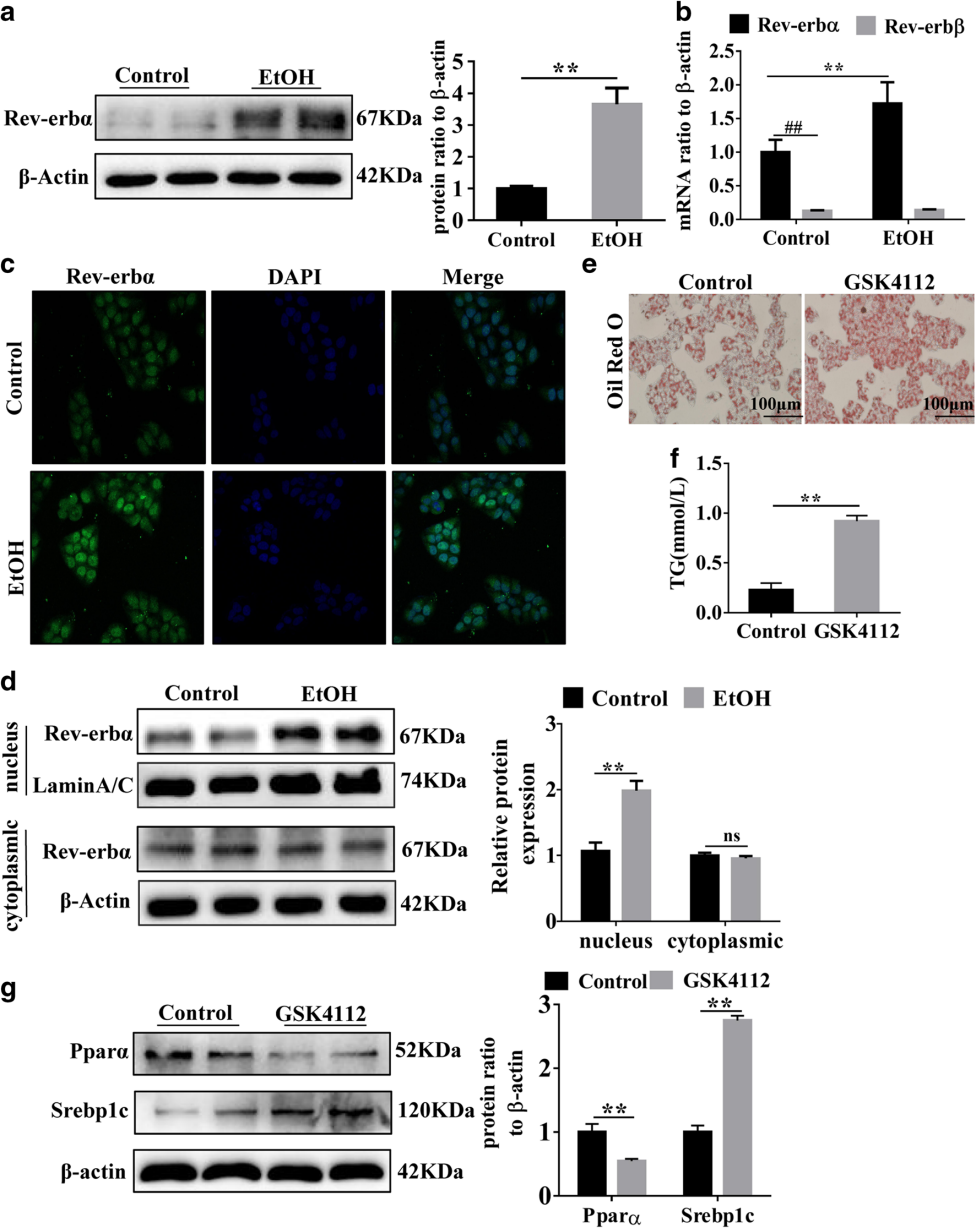
Rev-erbα was up-regulated in EtOH-treated L-02 cells and mediated liver steatosis in vitro*.* L-02 cells were treated with 150 mM EtOH for 48 h. **A** Western blot analysis of Rev-erbα expression in Control and EtOH group. **B** qRT-PCR analysis of Rev-erbα and Rev-erbβ in Control and EtOH group. **C** Immunofluorescence analysis of nuclear localization of Rev-erbα in Control and EtOH group (scale bar = 40 μm). **D** Western blot analysis of Rev-erbα in nucleus and cytoplasm in control and EtOH group. L-02 cells were treated with or without 10 μM GSK4112 for 24 h. **E**, **F** ORO staining and TG content analysis in control and GSK4112 group (scale bar = 100 μm). **G** Western blot analysis of Pparα and Srebp1c in control and GSK4112 group (n = 3). Bar represents the mean ± SEM. Significance *P < 0.05, **P < 0.01 vs. control group, ^#^*P* < 0.05, ^##^*P* < 0.01 vs. Rev-erbα in control group

In vitro, L-02 cells were treated with EtOH (150 mM, 48 h). As shown in Additional file 1: Fig. S1A, B, lipid droplets and the level of TG were significantly increased in EtOH-treated cells compared to L-02 cells. Then, qRT-PCR analysis revealed that the expression of Pparα was decreased and Srebp1c was increased in EtOH-treated L-02 cells compared to L-02 cells, this result was further confirmed by western blot analysis (Additional file 1: Fig. S1C, D). The above evidence showed that 150 mM EtOH could cause disorder of lipid metabolism in L-02 cells. Consistenting with results in vivo, the mRNA expression of Rev-erbα was higher than Rev-erbβ in L-02 cells, and Rev-erbα was increased while Rev-erbβ had no obvious change in EtOH-treated L-02 cells (Fig. 2B). Western blot analysis further confirmed that Rev-erbα was increased in EtOH-treated L-02 cells (Fig. 2A). Furthermore, immunofluorescence was used to detect intracellular distribution of Rev-erbα in L-02 cells, the result indicated that Rev-erbα was significantly elevated in the nucleus but almost unchanged in the cytoplasm after treatment with EtOH for 48 h in L-02 cells, this founding was further demonstrated by western blot analysis (Fig. 2C, D).

Steatosis is the main pathological process of AFL [5, 6]. Studies have shown that Rev-erbα can regulate lipid metabolism [10, 11]. To investigate whether Rev-erbα is involved in liver steatosis, L-02 cells were treated with Rev-erbα agonist GSK4112 at a fixed time of the day, at 5 p.m. (10 μM, 24 h) [23, 24]. Compared with L-02 cells, lipid droplets and TG level were significantly increased, and the protein of Pparα was down-regulated but Srebp1c was up-regulated in GSK4112-treated L-02 cells (Fig. 2E–G). These results indicated that activation of Rev-erbα can induce disorder of lipid metabolism and Rev-erbα may be involved in the pathological process of AFL.

### SR8278 attenuates steatosis in the liver of EtOH-fed mice and EtOH-treated L-02 cells

**Fig. 3:**
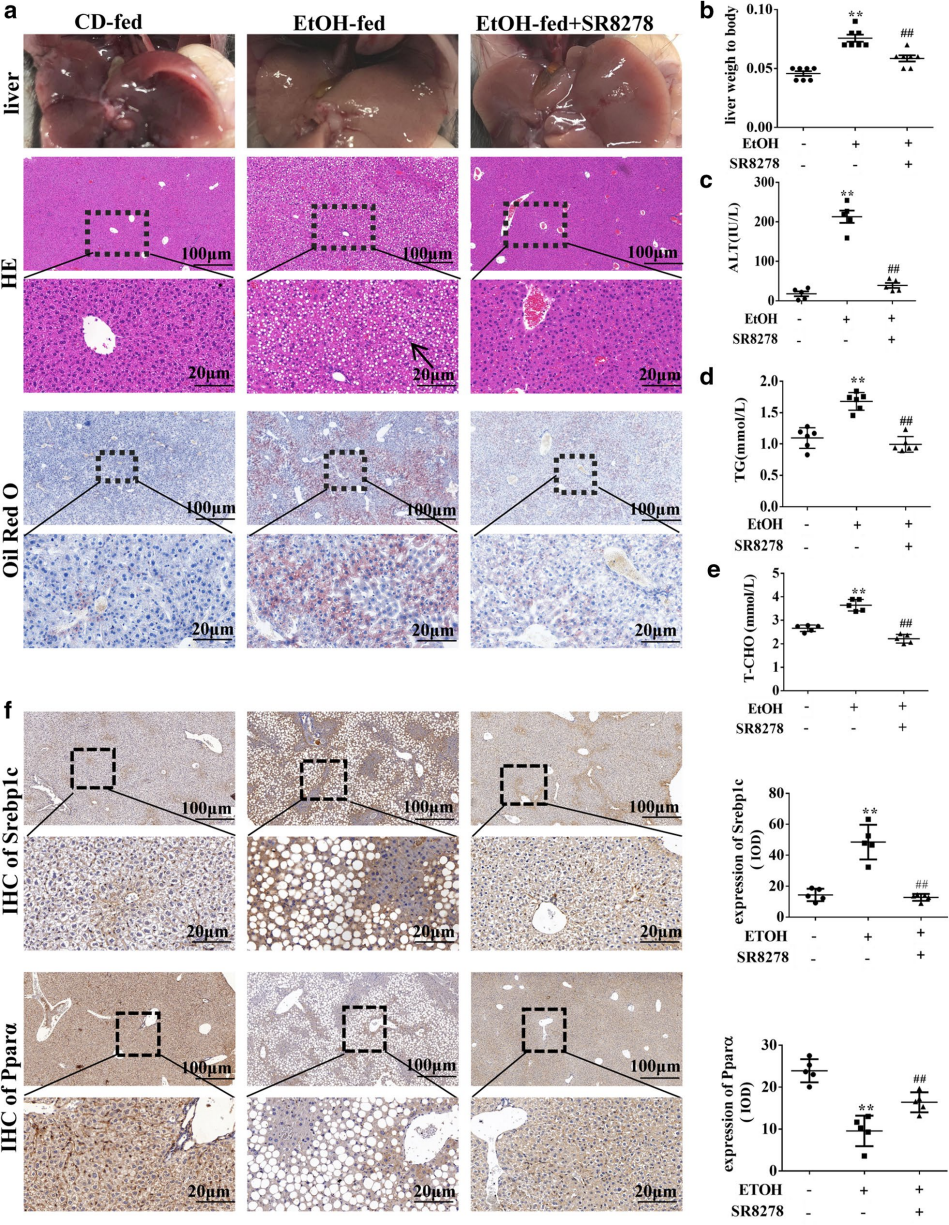
SR8278 (Rev-erbα antagonist) attenuates steatosis in the liver of EtOH-fed mice and EtOH-treated L-02 cells. Mice fed EtOH liquid were tail vein injection with SR8278 (2 mg/kg) for 3 days. **A** Representative image of liver and histological assessment of hepatic pathologic alterations by H&E and ORO staining in the liver of mice with or without SR8278 injection (scale bar = 100 μm or 20 μm). **B**–**E** The liver weight ratio to body of mice, serum ALT, TG level and TC levels in the liver of mice. **F** The immunohistochemistry staining of Pparα and Srebp1c in the liver of mice (scale bar = 100 μm or 20 μm) (n = 6). Bar represents the mean ± SEM. Significance *P < 0.05, **P < 0.01 vs. CD-fed group. ^#^*P* < 0.05, ^##^*P* < 0.01 vs. EtOH-fed group

To better understand the function of Rev-erbα in EtOH-induced liver injury and steatosis, the Rev-erbα antagonist SR8278 (2 mg/kg) was injected in EtOH-fed mice via tail vein, half an hour after feeding [25–27]. As shown in Fig. 3A, the fatty liver was significantly alleviated in EtOH-fed mice after injecting SR8278 for 3 days. H&E and ORO staining revealed interlobular space of liver, inflammatory cell infiltration and lipid droplets were improved after treatment with SR8278. The ratio of liver to body weight was increased and serum ALT, TG, and T-CHO levels in EtOH-fed mice were decreased by SR8278 (Fig. 3B–E). Moreover, increased of Pparα and decreased of Srebp1c were showed by immunohistochemistry analysis (Fig. 3F).

Additionally, SR8278 (the antagonist of Rev-erbα, 10 μM) reduced the level of TG and intracellular lipid droplets in EtOH-treated L-02 cells for 24 h (Additional file 1: Fig. S1E, F), and higher expression of Pparα and lower expression of Srebp1c were demonstrated by western blot analysis (Additional file 1: Fig. S1G).

### Down-regulation of Rev-erbα attenuates steatosis in EtOH-treated L-02 cells

**Fig. 4:**
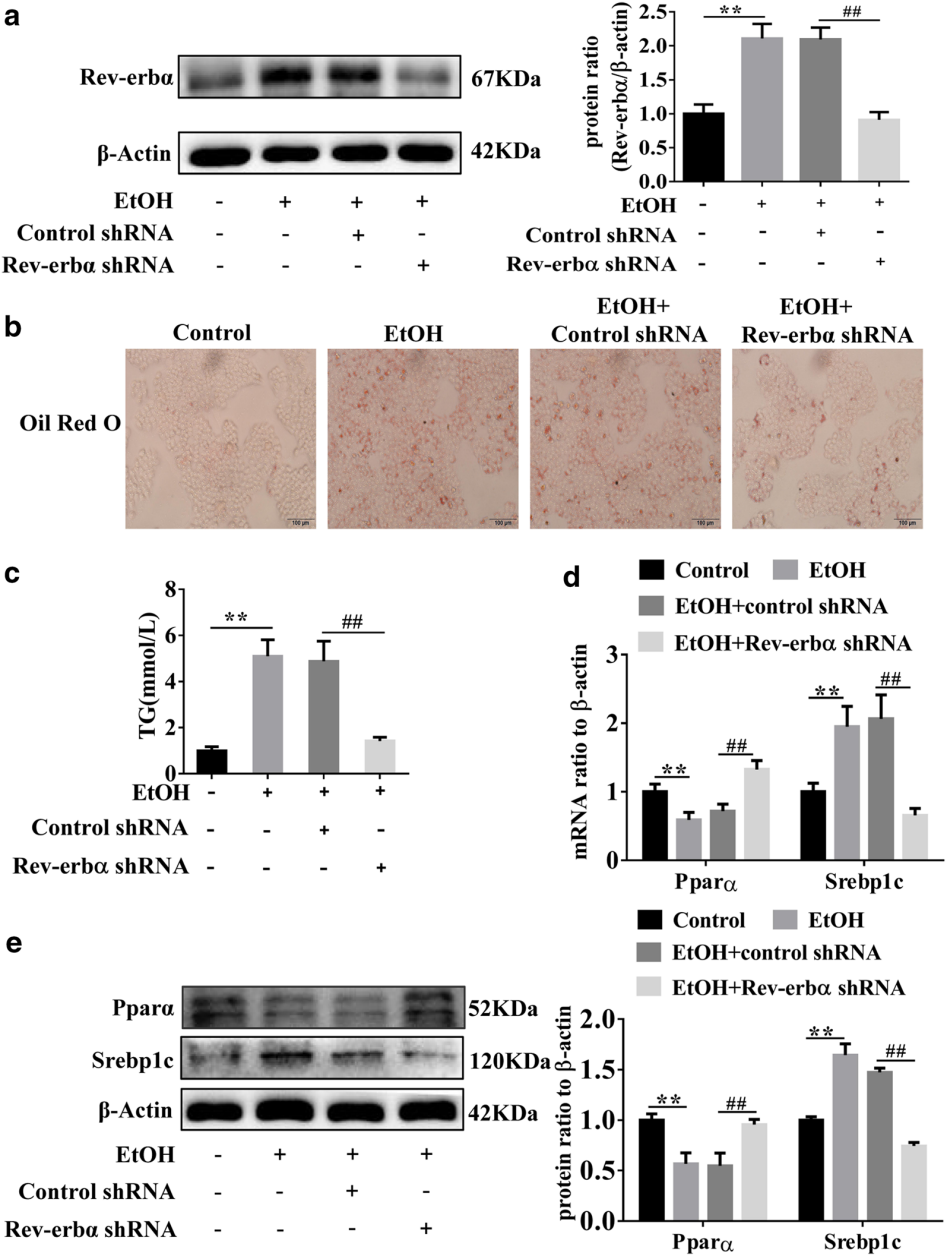
Down-regulated Rev-erbα attenuates steatosis in the EtOH-treated L-02 cells. **A** Western blot analysis of Rev-erbα in EtOH-treated L-02 cells transfected with Rev-erbα shRNA. **B**, **C** ORO staining and TG content analysis in EtOH-treated L-02 cells after Rev-erbα knockout (magnification ×200, scale bar = 100 μm). **D**, **E** Western blot and qRT-PCR analysis of Pparα and Srebp1c in EtOH-treated L-02 cells transfected with Rev-erbα shRNA (n = 3). Bar represents the mean ± SEM. Significance *P < 0.05, **P < 0.01 vs. control group. ^#^*P* < 0.05, ^##^*P* < 0.01 vs. control shRNA group

To further verify the effect of Rev-erbα on lipid metabolism, Rev-erbα was silenced down by transfecting Rev-erbα shRNA in L-02 cells. The results of qRT-PCR and western blot showed that Rev-erbα was knocked down by Rev-erbα shRNA in EtOH-treated L-02 cells (Additional file 1: Fig. S1H and Fig. 4A). Then, the results of ORO staining, TG assay and the levels of Pparα and Srebp1c showed that Rev-erbα shRNA improved lipid metabolism disorder and reduced lipid deposition (Fig. 4B, E). In summary, inhibition or silencing of Rev-erbα may attenuate steatosis in vivo and in vitro*.*

### Rev-erbα regulates lipid metabolism by enhancing autophagy activity in vivo and in vitro

**Fig. 5:**
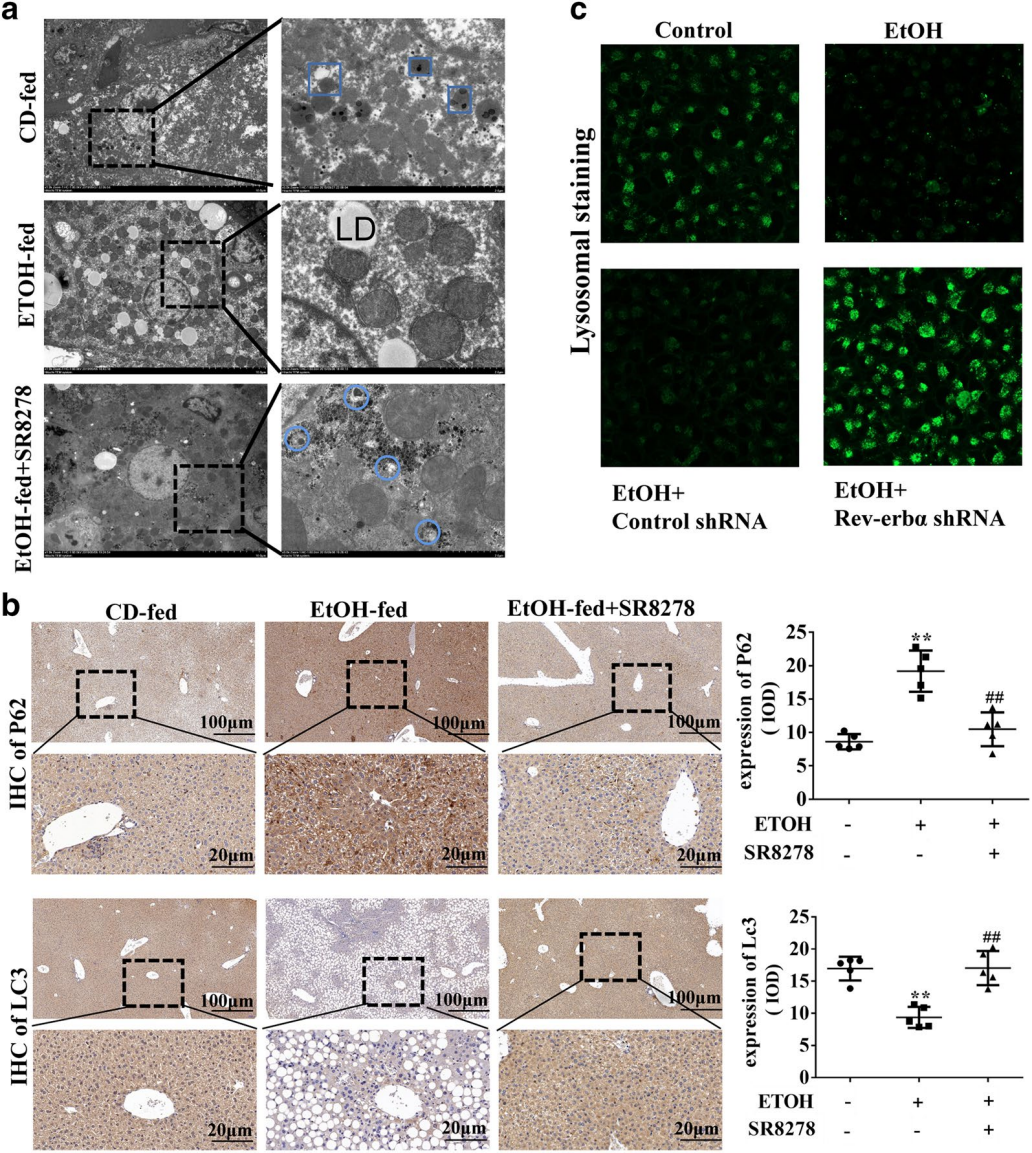
Rev-erbα regulated lipid metabolism by enhancing autophagy activity in vivo and vitro. **A** Electron microscopy of liver tissues of EtOH fed mice injected with or without SR8278 at 10 μm and at 2 μm (*N* nucleus, *M* mitochondria, *LD* lipid droplet; triangle refers to autophagy; arrow refers to lysosome) (n ≥ 6). **B** Histopathological analysis of P62 and Lc3 by immunohistochemistry in the liver of EtOH-fed mice injected with or without SR8278 (scale bar = 100 μm or 20 μm). Bar represents the mean ± SEM. Significance *P < 0.05, **P < 0.01 vs. CD-fed group. ^#^*P* < 0.05, ^##^*P* < 0.01 vs. EtOH-fed group. **C** Lysosome staining with Lyso Tracter Green DND-26 in EtOH-treated L-02 cells transfected with Rev-erbα shRNA (scale bars = 40 μm) (n ≥ 3). Bar represents the mean ± SEM. Significance *P < 0.05, **P < 0.01 vs. control group. ^#^*P* < 0.05, ^##^*P* < 0.01 vs. control shRNA group

It is well known that autophagy is involved in the degradation of lipid droplets and regulation of lipid metabolism [16, 17]. Rev-erbα has been reported to regulate autophagy in skeletal muscle [18, 19]. Therefore, we hypothesized that Rev-erbα could ameliorate EtOH-induced lipid steatosis by regulating autophagy. As shown in Fig. 5A, electronic microscopy showed that autophagosome and lysosomal were significantly decreased by EtOH, and SR8278 could increase the number of autophagosome and lysosomal in EtOH-fed mice. Immunohistochemistry analysis further showed that SR8278 increased the expression of Lc3 and decreased the expression of P62 in EtOH-fed mice (Fig. 5B).

In vitro, Lyso Tracker Green DND-26 analysis showed that lysosomal acidity was decreased by EtOH in L-02 cells while it was increased in EtOH-treated L-02 cells by SR8278 and Rev-erbα shRNA (Additional file 1: Fig. S2A and Fig. 5C). Next, western blot analysis confirmed that compared to L-02 cells, the ratio of LC3II/I and the level of Beclin1 were decreased but P62 was increased in EtOH-treated L-02 cells, however, the results were reversed after treatment with SR8278 and Rev-erbα shRNA (Additional file 1: Fig. S2B, C). Above experimental results indicated that autophagy can be negatively regulated by Rev-erbα in AFL.

### Rev-erbα inhibits the activity of autophagy through regulating Bmal1

**Fig. 6:**
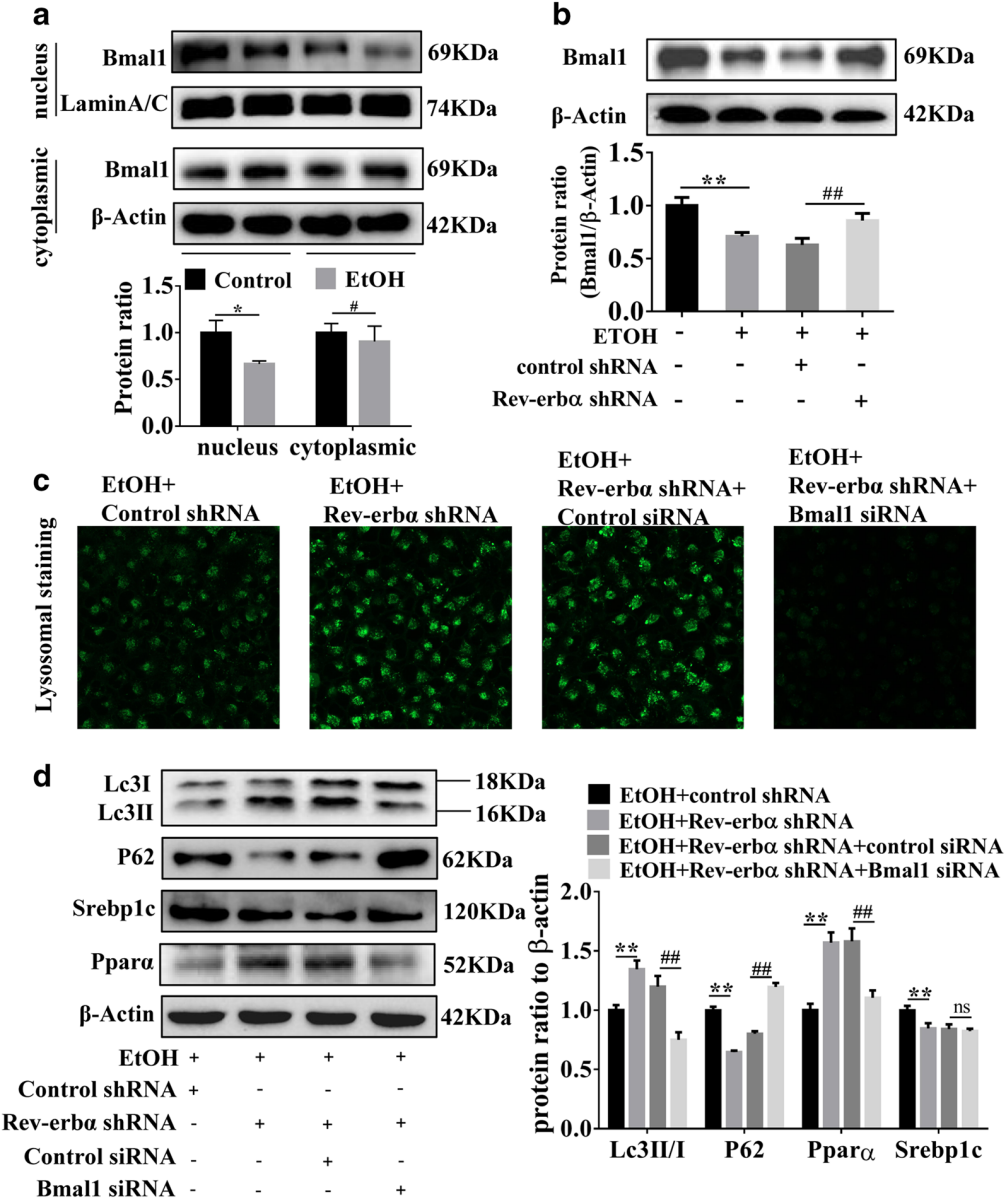
Rev-erbα inhibits the activity of autophagy through regulating Bmal1. **A** Western blot analysis of Bmal1 in nucleus and cytoplasm in EtOH-treated L-02 cells. Bar represents the mean ± SEM. Significance *P < 0.05, **P < 0.01 vs. EtOH-fed group or EtOH group. **B** Western blot analysis of Bmal1 in EtOH-treated L-02 cells transfected with Rev-erbα shRNA. Bar represents the mean ± SEM. Significance *P < 0.05, **P < 0.01 vs. control group, ^#^*P* < 0.05, ^##^*P* < 0.01 vs. control shRNA group. **C** Lysosome staining by using Lyso Tracter Green DND-26 (50 nM) in EtOH-treated L-02 cells transfected with Bmal1 siRNA and Rev-erbα shRNA (n = 3) (scale bars = 40 μm). **D** Western blot analysis of Lc3II/I, P62, Pparα and Srebp1c in EtOH-treated L-02 cells transfected with Bmal1 siRNA and Rev-erbα shRNA. Bar represents the mean ± SEM. Significance *P < 0.05, **P < 0.01 vs. EtOH + control shRNA group, ^#^*P* < 0.05, ^##^*P* < 0.01 vs. EtOH + Rev-erbα shRNA + control siRNA group

As presented in Additional file 1: Fig. S3A, B, the level of Bmal1 was decreased in the liver of EtOH-fed mice and EtOH-treated L-02 cells. Moreover, Bmal1 protein was decreased prominently in the nucleus and had no difference in the cytoplasm in EtOH-treated L-02 cells compared to L-02 cells (Fig. 6A). Knockdown of Rev-erbα up-regulated the expression of Bmal1 detected by using western blot and qRT-PCR analysis (Fig. 6B and Additional file 1: Fig. S3C). The above experimental results showed that Rev-erbα might play a critical role in regulating the expression of Bmal1 in AFL.

To further confirm whether Rev-erbα inhibit autophagy dependent Bmal1 in AFL, Rev-erbα shRNA and Bmal1 siRNA were co-transfected into EtOH-treated L-02 cells. First, the results of qRT-PCR and western blot showed that Bmal1 was knocked down by Bmal1 siRNA in EtOH treatment L-02 cells (Additional file 1: Fig. S3D, E). As illustrated in Fig. 6C, Bmal1 siRNA have reversed the acidity of lysosomes which was increased by Rev-erbα shRNA in EtOH treatment L-02 cells. What’s more, western blot analysis showed that Rev-erbα shRNA and Bmal1 siRNA co-transfection decreased the ratio of LC3 II/I, increased P62 level, and downregulated Pparα in EtOH treatment L-02 cells but Srebp1c has no significantly changed (Fig. 6D). These data indicated that Rev-erbα may inhibit the activity of autophagy by Bmal1 in EtOH-induced lipid steatosis.

## Discussion

Hepatocytes lipid accumulation is a typical morphological characteristic of AFL [6, 28]. According to the initial ‘two hit hypothesis,’ inflammation as the second hit promotes the transformation from AFL to ASH on the base of liver steatosis [29]. As an important node in the development of AFLD, the improvement of hepatic lipid in AFL prevent the occurrence of ASH and reduce the incidence of ALD. The nuclear receptor Rev-erbs are known to regulate multiple downstream genes involved in diverse cellular functions including metabolism, widely participating in the physiological process of energy, glucose and lipid metabolism [7–12]. However, the effect of Rev-erbs on lipid regulation in alcoholic fatty liver remains to be studied. Rev-erbs has two subtypes with high homology and their distribution are different [30–35]. In this study, we found that Rev-erbα has a more abundant distribution compared to Rev-erbβ in the liver of mice and L-02 cells, this result was consistent with the research that Rev-erbα was higher in liver, meanwhile Rev-erbβ was lower in physiological system but higher in CNS in mice [36]. In addition, we showed higher expression of Rev-erbα in vitro and in vivo accompanying with lipid droplets and triglycerides. Moreover, L-02 cells showed significant steatosis when cells were treated with GSK4112. These results indicate that Rev-erbα is closely related to liver lipid metabolism. Importantly, steatosis was ameliorated in the liver of EtOH-fed mice after the mice were treated with SR8278, the same result was found when EtOH-treated L-02 cells were treated with SR8278 or transfected with Rev-erbα ShRNA. Rev-erbα is a member of nuclear receptor superfamily and plays an important role in transcriptional inhibition [37–42]. We found that Rev-erbα was mainly up-regulated in the nucleus in EtOH-treated L-02 cells. Given that Rev-erbα is a nuclear transcription factor, we speculate that Rev-erbα may play a transcriptional regulatory role in lipid regulation. Coincidentally, Srebp1c was decreased and pparα was increased at both mRNA and protein levels in SR8278 treated EtOH-fed mice and SR8278/Rev-erbα ShRNA treated EtOH-treated L-02 cells. To sum up, Rev-erbα may be a vital mediator of lipid metabolism in vivo and vitro*.*

Autophagy is a wide range of cells and lysosomal-dependent degradation pathway. It is a mechanism that delivers cytoplasmic cargo into acidic compartments of the cell known as lysosomes. Acidic environment in lysosomes is the key to autophagy [43, 44]. Singh et al. identified that autophagy was required for lipid droplets breakdown and inhibition of autophagy increases lipid storage [45, 46]. Other studies, for example, Martinez-Lopez et al. reported that cold triggered lipolysis by inducing autophagy in mouse liver [47]. In this study, the ratio of autophagosomes membrane protein LC3II/I was downregulated. Furthermore, the acidity in lysosomes was decreased and the level of p62 was increased significantly in the liver of EtOH-fed mice and EtOH-treated L-02 cells. These studies indicated that the formation of autophagosomes and lysosomal function was impaired in vivo and vitro. What's intriguing is that the activity of autophagy accompanying by increasing lysosomal acidity was promoted by reducing Rev-erbα. In summary, our works have shed light on an enhanced autophagosome and lysosomal function through normalizing Rev-erbα expression in AFL.

Previous studies have found that Bmal1 (aryl-hydrocarbon nuclear translocator-like 1) drives the cyclic expression genes involved in lipid metabolism [48]. Zhang et al. has reported that mice with Bmal1 depletion were more susceptible to ethanol induced fatty liver and liver injury while Bmal1 over-expression protects EtOH-fed mice from fatty liver and liver injury [49]. Taking Rev-erbα plays a negative adjustment function through inhibits Bmal1 transcription by target a ROR-response element in the promoter of the Bmal1 gene into consideration [37, 38], we speculate that Rev-erbα may interact with Bmal1 in cell metabolism. Indeed, we observed that Bmal1 was decreased in the nucleus in EtOH-treated L-02 cells, this result is consistent with Rev-erbα in nucleus. Further, Bmal1 was increased by Rev-erbα inhibition in EtOH-treated L-02 cells, which suggested that there may be a crosstalk between Rev-erbα and Bmal1 in the nucleus respond to ethanol treatment. Importantly, the activity of autophagy was inhibited by Bmal1 depletion, as well as the promotion of Rev-erbα on Pparα expression was abolished. It is likely that the modest suppression of Rev-erbα promoted Pparα-dependent β-oxidation pathway by up-regulating Bmal1 through regulating autophagy in EtOH-treated L-02 cells. It is well known that Pparα and Srebp1c plays a pivotal role in lipid metabolism-associated transcription factors [5, 50, 51]. However, Bmal1 knockdown did not change the positive regulatory effect of Rev-erbα on Srebp1c. Previous study has demonstrated Rev-erbα inhibits transcription mainly by recruiting HDAC3 and NCoR. But Srebp1c expression is not HDAC3-sensitive, Berthier et al. has proved that Rev-erbα directly bound to genomic regions in the vicinity or within the Srebf1 gene [52]. These data suggest that Rev-erbα may be directly involved in the expression of lipid synthesis genes, which is independent of Bmal1 expression.

**Fig. 7:**
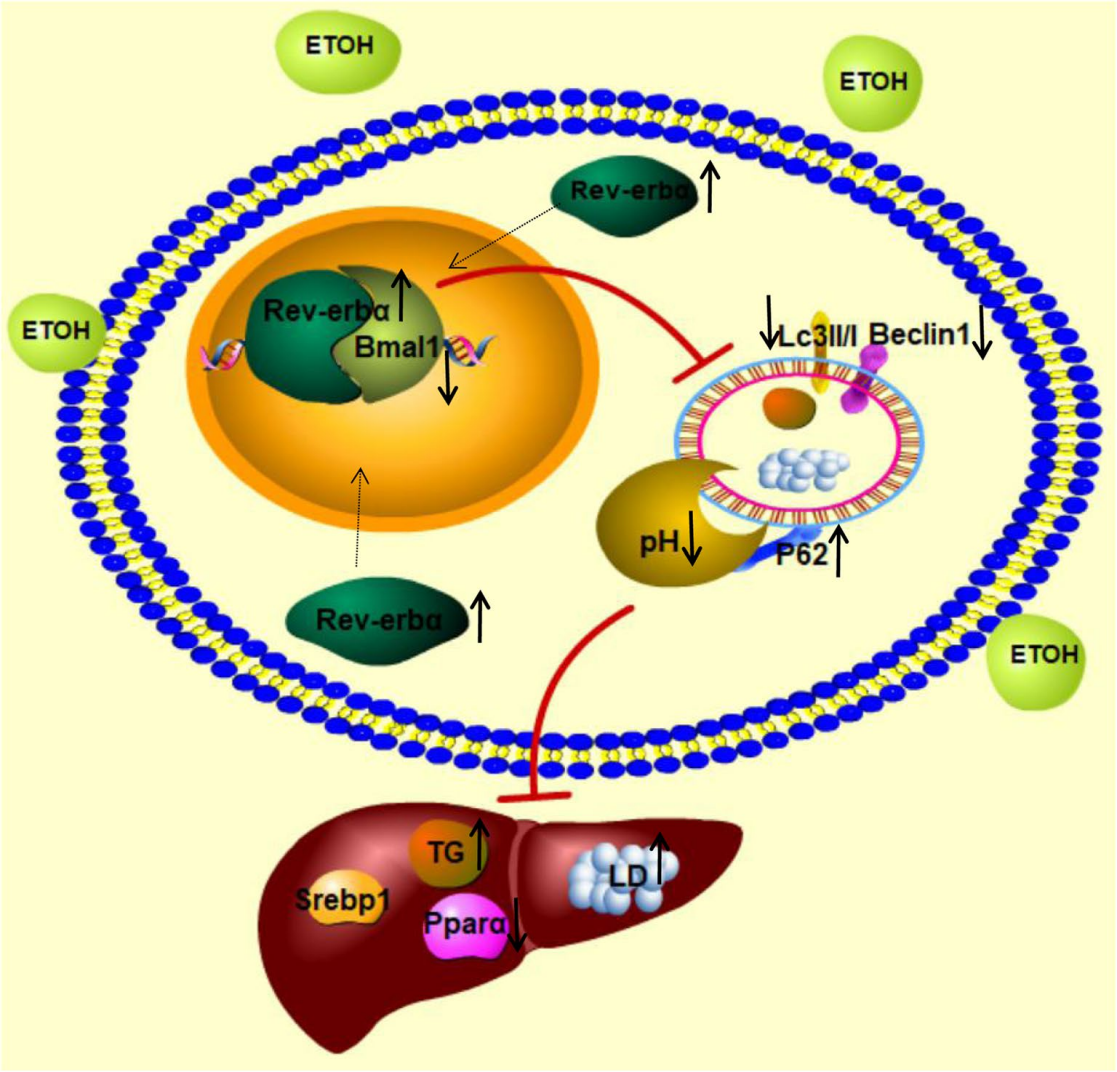
Schematic diagram of the molecular mechanism of Rev-erbα-induced lipid steatosis in the liver. Rev-erbα was enhanced by EtOH and bound to the Bmal1 promoter to regulated the activity of autophagy to regulated lipid metabolism

In conclusion, we presented evidence supporting that Rev-erbα was higher expressed and reducing Rev-erbα could improve the accumulation of lipid in vivo and vitro. Impaired autophagy function in the liver of EtOH-fed mice and EtOH-treated L-02 cells was enhanced by limiting the activity of Rev-erbα. Further studies promulgated a regulation of Rev-erbα on autophagy by Bmal1 thus influencing hepatic fatty acid oxidation pathway in vitro (Fig. 7). Our findings identified a critical role of Rev-erbα in ameliorating lipid metabolism through autophagy by impacting on Bmal1. It suggests that focusing on Rev-erbα function in lipid metabolism may offer a therapeutic approach to AFL. The biggest flaw in our research is that Rev-erbα and Bmal1 were only used as the regulatory factors of lipid metabolism, the changes of their biological rhythm were not studied, which needs to be improved.

## Conclusion

Our observations emphasize an important role of Rev-erbα that may promote the progress of liver steatosis and functioned as a negative regulator of autophagy. Subsequently, we demonstrate that Rev-erbα regulates autophagy in a Bmal1 dependent manner. This study provides scientific basis for targeted therapy of AFL.

## Supplementary Information

**Additional file 1: Figure S1.** 150 mmol/L EtOH incubation induces steatosis in L02 cells and steatosis was ameliorated by SR8278 treatment or Rev-erbαshRNA transfection in EtOH-treated L-02 cells. **Figure S2.** Autophagy activity was improved by SR8278 treatment or Rev-erbαshRNA transfection in EtOH-treated L-02 cells. **Figure S3.** Bmal1 was up-regulated in EtOH-treated L-02 cells and was regulated by Rev-erbα.

## Data Availability

All supporting data included in the main article and its supplementary files are available from the corresponding author upon request.

## Abbreviations

AFLD: Alcoholic fatty liver
ALD: Alcoholic liver disease
AFL: Alcoholic fatty liver
ASH: Alcoholic steatohepatitis
AF: Alcoholic fibrosis
AC: Alcoholic cirrhosis
AHCC: Alcoholic hepatocellular carcinoma
Rev-erbα: Nuclear receptor subfamily 1 group D member 1
Rev-erbβ: Nuclear receptor subfamily 1 group D member2
Pparα: Peroxisome proliferators activated receptor α
Srebp1c: Sterol regulatory element binding protein-1
LC3: Microtubule-associated light chain 3
Beclin1: Programmed cell death-1
P62: Multifunctional adaptor protein
Bmal1: Brain and muscle aryl hydrocarbon receptor nuclear translocator (ARNT)-like protein-1
ALT: Alanine aminotransferase
TG: Triglyceride
T-CHO: Total cholesterol
SR8278: (*S*)-Ethyl 2-(5-(methylthio) thiophene-2-carbonyl)-1,2,3,4-tetrahydroisoquinoline-3-carboxylate
GSK-4112: 1,1-Dime-thylethyl-*N*-[(4-chorophenyl)]-*N*-[(5-nitr*o*-2-thienyl])-glycinate
siRNA: Small interfering RNA
shRNA: Short hairpin RNA

## References

[1] Rehm J, Samokhvalov AV, Shield KD (2013). Global burden of alcoholic liver diseases. J Hepatol.

[2] European Association for the Study of Liver (2012). EASL clinical practical guidelines: management of alcoholic liver disease. J Hepatol.

[3] O’Shea RS, Dasarathy S, McCullough AJ (2010). Alcoholic liver disease. Hepatology.

[4] Friedman SL, Neuschwander-Tetri BA, Rinella M, Sanyal AJ (2018). Mechanisms of NAFLD development and therapeutic strategies. Nat Med.

[5] Liu J (2014). Ethanol and liver: recent insights into the mechanisms of ethanol-induced fatty liver. World J Gastroenterol.

[6] You M, Fischer M, Deeg MA, Crabb DW (2002). Ethanol induces fatty acid synthesis pathways by activation of sterol regulatory element-binding protein (SREBP). J Biol Chem.

[7] Altman BJ, Hsieh AL, Gouw AM, Dang CV (2017). Correspondence: oncogenic MYC persistently upregulates the molecular clock component REV-ERBα. Nat Commun.

[8] Wang J, Yin L, Lazar MA (2006). The orphan nuclear receptor Rev-erb alpha regulates circadian expression of plasminogen activator inhibitor type 1. J Biol Chem.

[9] Li T, Eheim AL, Klein S (2014). Novel role of nuclear receptor Rev-erbα in hepatic stellate cell activation: potential therapeutic target for liver injury. Hepatology.

[10] Wang X, Wang N, Wei X, Yu H, Wang Z (2018). REV-ERBα reduction is associated with clinicopathological features and prognosis in human gastric cancer. Oncol Lett.

[11] Delezie J, Dumont S, Dardente H (2012). The nuclear receptor REV-ERBα is required for the daily balance of carbohydrate and lipid metabolism. FASEB J.

[12] Kan HY, Georgopoulos S, Zannis VA (2000). Hormone response element in the human apolipoprotein CIII (ApoCIII) enhancer is essential for intestinal expression of the ApoA-I and ApoCIII genes and contributes to the hepatic expression of the two linked genes in transgenic mice. J Biol Chem.

[13] Fontaine C, Dubois G, Duguay Y (2003). The orphan nuclear receptor Rev-Erbalpha is a peroxisome proliferator-activated receptor (PPAR) gamma target gene and promotes PPARgamma-induced adipocyte differentiation. J Biol Chem.

[14] Sitaula S, Zhang J, Ruiz F, Burris TP (2017). Rev-erb regulation of cholesterologenesis. Biochem Pharmacol.

[15] Singh R, Kaushik S, Wang Y, Zhong Z, Sanchez-Lopez E (2016). Autophagy, inflammation, and immunity: a troika governing cancer and its treatment. Cell.

[16] Stolz A, Ernst A, Dikic I (2014). Cargo recognition and trafficking in selective autophagy. Nat Cell Biol.

[17] Saito T, Kuma A, Sugiura Y (2019). Autophagy regulates lipid metabolism through selective turnover of NCoR1. Nat Commun.

[18] Mayeuf-Louchart A, Thorel Q, Delhaye S (2017). Rev-erb-α regulates atrophy-related genes to control skeletal muscle mass. Sci Rep.

[19] Woldt E, Sebti Y, Solt LA (2013). Rev-erb-α modulates skeletal muscle oxidative capacity by regulating mitochondrial biogenesis and autophagy. Nat Med.

[20] Grimaldi B (2015). Lysosomotropic REV-ERB antagonism: a metabolic connection between circadian rhythm and autophagy may tell cancer cells “it's time to die”. Mol Cell Oncol.

[21] Huang G, Zhang F, Ye Q (2016). The circadian clock regulates autophagy directly through the nuclear hormone receptor Nr1d1/Rev-erbα and indirectly via Cebpb/(C/ebpβ) in zebrafish. Autophagy.

[22] Bertola A, Mathews S, Ki SH, Wang H, Gao B (2013). Mouse model of chronic and binge ethanol feeding (the NIAAA model). Nat Protoc.

[23] Chen H, Chu G, Zhao L (2012). Rev-erbα regulates circadian rhythms and StAR expression in rat granulosa cells as identified by the agonist GSK4112. Biochem Biophys Res Commun.

[24] Grant D, Yin L, Collins JL (2010). GSK4112, a small molecule chemical probe for the cell biology of the nuclear heme receptor Rev-erbα. ACS Chem Biol.

[25] Dong D, Sun H, Wu Z, Wu B, Xue Y, Li Z (2016). A validated ultra-performance liquid chromatography-tandem mass spectrometry method to identify the pharmacokinetics of SR8278 in normal and streptozotocin-induced diabetic rats. J Chromatogr B Analyt Technol Biomed Life Sci.

[26] Welch RD, Billon C, Valfort AC, Burris TP, Flaveny CA (2017). Pharmacological inhibition of REV-ERB stimulates differentiation, inhibits turnover and reduces fibrosis in dystrophic muscle. Sci Rep.

[27] Kojetin D, Wang Y, Kamenecka TM, Burris TP (2011). Identification of SR8278, a synthetic antagonist of the nuclear heme receptor REV-ERB. ACS Chem Biol.

[28] Shimano H, Horton JD, Hammer RE, Shimomura I, Brown MS, Goldstein JL (1996). Overproduction of cholesterol and fatty acids causes massive liver enlargement in transgenic mice expressing truncated SREBP-1a. J Clin Invest.

[29] Filiano AN, Millender-Swain T, Johnson R, Young ME, Gamble KL, Bailey SM (2013). Chronic ethanol consumption disrupts the core molecular clock and diurnal rhythms of metabolic genes in the liver without affecting the suprachiasmatic nucleus. PLoS ONE.

[30] Le Martelot G, Claudel T, Gatfield D (2009). REV-ERBalpha participates in circadian SREBP signaling and bile acid homeostasis. PLoS Biol.

[31] Feng D, Liu T, Sun Z (2011). A circadian rhythm orchestrated by histone deacetylase 3 controls hepatic lipid metabolism. Science.

[32] Bugge A, Feng D, Everett LJ (2012). Rev-erbα and Rev-erbβ coordinately protect the circadian clock and normal metabolic function. Genes Dev.

[33] Solt LA, Wang Y, Banerjee S (2012). Regulation of circadian behaviour and metabolism by synthetic REV-ERB agonists. Nature.

[34] Gagnidze K, Hajdarovic KH, Moskalenko M, Karatsoreos IN, McEwen BS, Bulloch K (2016). Nuclear receptor REV-ERBα mediates circadian sensitivity to mortality in murine vesicular stomatitis virus-induced encephalitis. Proc Natl Acad Sci USA.

[35] Crumbley C, Wang Y, Kojetin DJ, Burris TP (2010). Characterization of the core mammalian clock component, NPAS2, as a REV-ERBalpha/ROR alpha target gene. J Biol Chem.

[36] Preitner N, Damiola F, Lopez-Molina L (2002). The orphan nuclear receptor REV-ERBalpha controls circadian transcription within the positive limb of the mammalian circadian oscillator. Cell.

[37] Curtis AM, Bellet MM, Sassone-Corsi P, O'Neill LA (2014). Circadian clock proteins and immunity. Immunity.

[38] Grimaldi B, Sassone-Corsi P (2007). Circadian rhythms: metabolic clockwork. Nature.

[39] Harding HP, Lazar MA (1993). The orphan receptor Rev-ErbA alpha activates transcription via a novel response element. Mol Cell Biol.

[40] Lazar MA, Hodin RA, Darling DS, Chin WW (1989). Novel member of the thyroid/steroid hormone receptor family is encoded by the opposite strand of the rat c-erbA alpha transcriptional unit. Mol Cell Biol.

[41] Kim YH, Marhon SA, Zhang Y, Steger DJ, Won KJ, Lazar MA (2018). Rev-erbα dynamically modulates chromatin looping to control circadian gene transcription. Science.

[42] Wagner M, Zollner G, Trauner M (2011). Nuclear receptors in liver disease. Hepatology.

[43] Liu CY, Wei XR, Chen Y (2019). Tetradecanuclear and octadecanuclear Gold(I) sulfido clusters: synthesis, structures, and luminescent selective tracking of lysosomes in living cells. Inorg Chem.

[44] Chao X, Ni H-M, Ding W-X (2018). Insufficient autophagy: a novel autophagic flux scenario uncovered by impaired liver TFEB-mediated lysosomal biogenesis from chronic alcohol-drinking mice. Autophagy.

[45] Singh R, Kaushik S, Wang Y (2009). Autophagy regulates lipid metabolism. Nature.

[46] Ward C, Martinez-Lopez N, Otten EG (2016). Autophagy, lipophagy and lysosomal lipid storage disorders. Biochim Biophys Acta.

[47] Martinez-Lopez N, Singh R (2015). Autophagy and lipid droplets in the liver. Annu Rev Nutr.

[48] Ma D, Li S, Molusky MM (2012). Circadian autophagy rhythm: a link between clock and metabolism?. Trends Endocrinol Metab.

[49] Zhang D, Tong X, Nelson BB (2018). The hepatic BMAL1/AKT/lipogenesis axis protects against alcoholic liver disease in mice via promoting PPARα pathway. Hepatology.

[50] Xu B, Jiang M, Chu Y (2018). Gasdermin D plays a key role as a pyroptosis executor of non-alcoholic steatohepatitis in humans and mice. J Hepatol.

[51] Papazyan R, Sun Z, Kim YH (2016). Physiological suppression of lipotoxic liver damage by complementary actions of HDAC3 and SCAP/SREBP. Cell Metab.

[52] Berthier A, Vinod M, Porez G (2018). Combinatorial regulation of hepatic cytoplasmic signaling and nuclear transcriptional events by the OGT/REV-ERBα complex. Proc Natl Acad Sci USA.

