# Supplementary material for: Rev-erbα exacerbates hepatic steatosis in alcoholic liver diseases through regulating autophagy
Source: Cell Biosci. 2021 Jul 10;11:129. doi: 10.1186/s13578-021-00622-4 (PMC8272374; doi:10.1186/s13578-021-00622-4)
Supplement: Supplementary file 1 — Additional file 1: Figure S1. 150 mmol/L EtOH incubation induces steatosis in L02 cells and steatosis was ameliorated by SR8278 treatment or Rev-erbαshRNA transfection in EtOH-treated L-02 cells. Figure S2. Autophagy activity was improved by SR8278 treatment or Rev-erbαshRNA transfection in EtOH-treated L-02 cells. Figure S3. Bmal1 was up-regulated in EtOH-treated L-02 cells and was regulated by Rev-erbα. [file 13578_2021_622_MOESM1_ESM.pdf]

Fig. S1

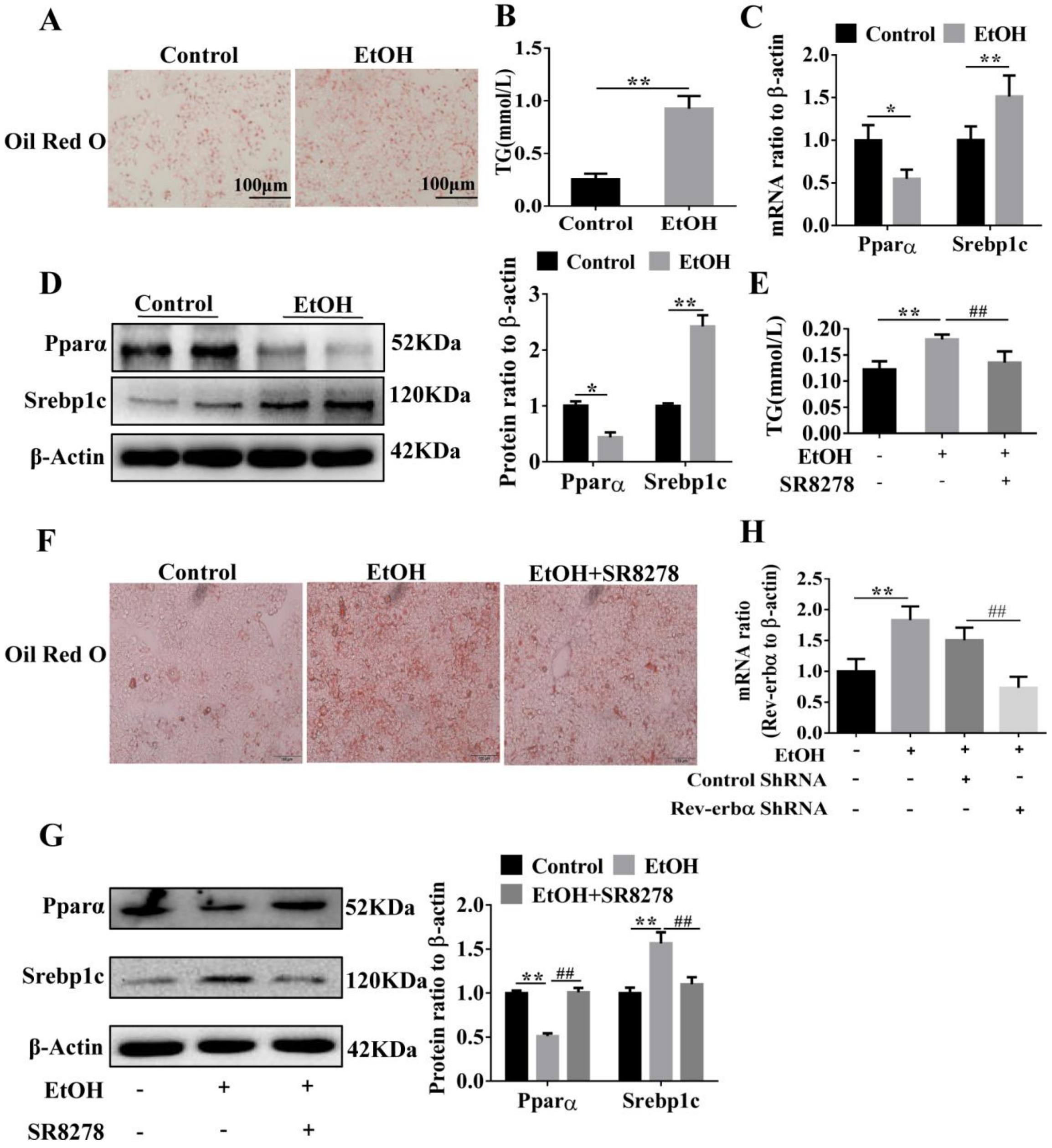

Figure S1. 150 mmol/L EtOH incubation induces steatosis in L02 cells and steatosis was ameliorated by SR8278 treatment or Rev-erbαshRNA transfection in EtOH-treated L-02 cells. (A) (B) Oil Red O staining and the Content of TG analysis in Control and EtOH group (Scale bar =100 μm). (C) (D) qRT-PCR and Western blot analysis of Pparα and Srebp1c in Control and EtOH group. EtOH-treated L-02 cells were treated with or without 10 μM SR8278 for 24 h. (E) (F) Oil Red O staining and TG content in EtOH-treated L-02 cells treated with SR8278 (Scale bar =100 μm). (G) Western blot analysis of Pparα and Srebp1c in EtOH-treated L-02 cells after SR8278 treatment. (H) qRT-PCR analysis of Rev-erbα in EtOH-treated L-02 cells transfected with Rev-erbαshRNA. (n=3). Bar represents the mean ± S.E.M.. Significance * $P < 0.05$, ** $P < 0.01$ vs. Control group. $^{\#}P < 0.05$, $^{\#\#}P < 0.01$ vs. EtOH group.

Fig. S2

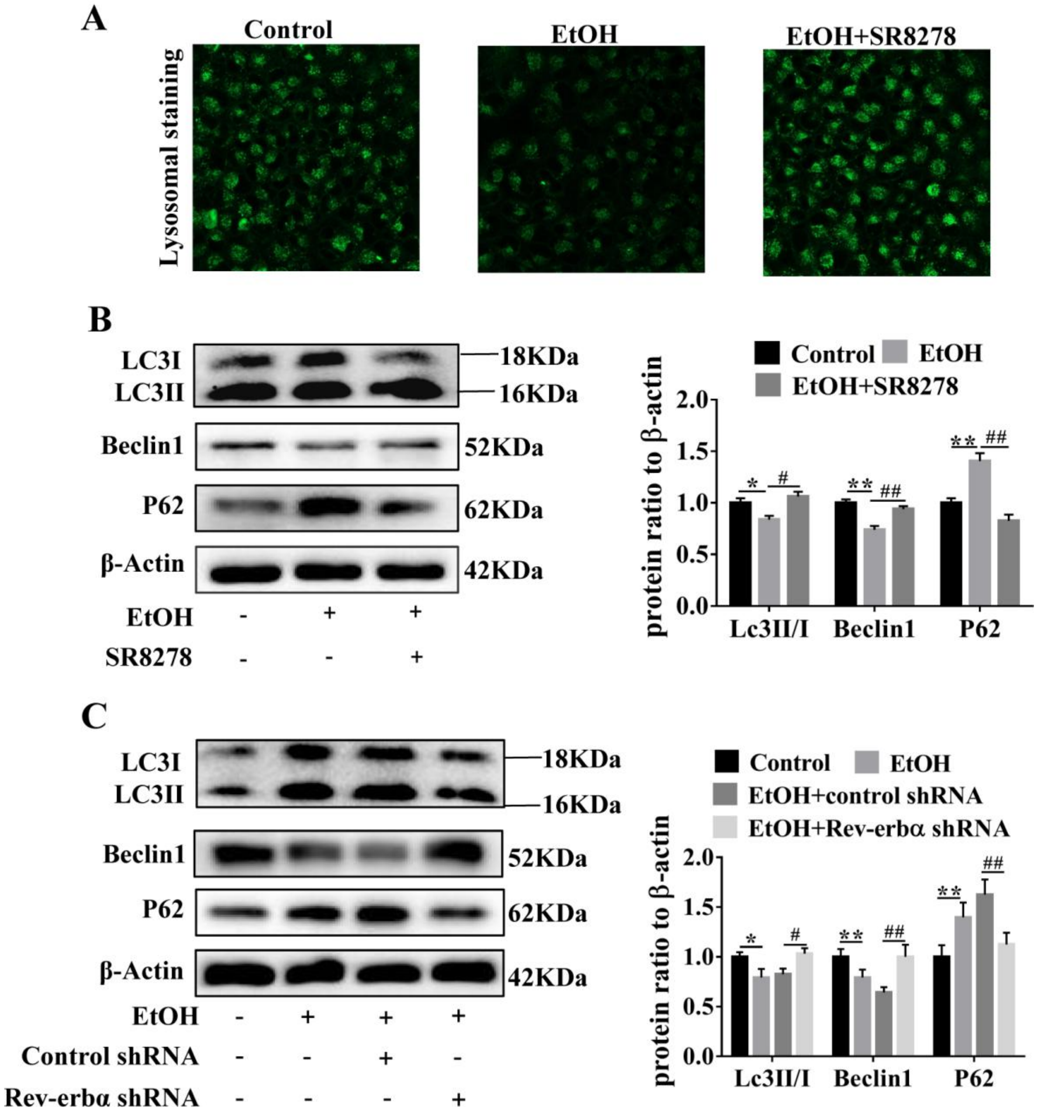

Figure S2. Autophagy activity was improved by SR8278 treatment or Rev-erbαshRNA transfection in EtOH-treated L-02 cells. (A) Lysosome staining with Lyso Tracter Green DND-26 in EtOH-treated L-02 cells treated with SR8278 (Scale bars=40 μm). (B) Western blot analysis of Lc3II/I, Beclin1 and P62 in EtOH-treated L-02 cells treated with SR8278. (C) Western blot analysis of Lc3II/I, Beclin1 and P62 in EtOH-treated L-02 cells in EtOH-treated L-02 cells transfected with Rev-erbα shRNA (n ≥3). Bar represents the mean ± S.E.M.. Significance * $P < 0.05$, ** $P < 0.01$ vs. Control group. # $P < 0.05$, ## $P < 0.01$ vs. Control shRNA group.

Fig. S3

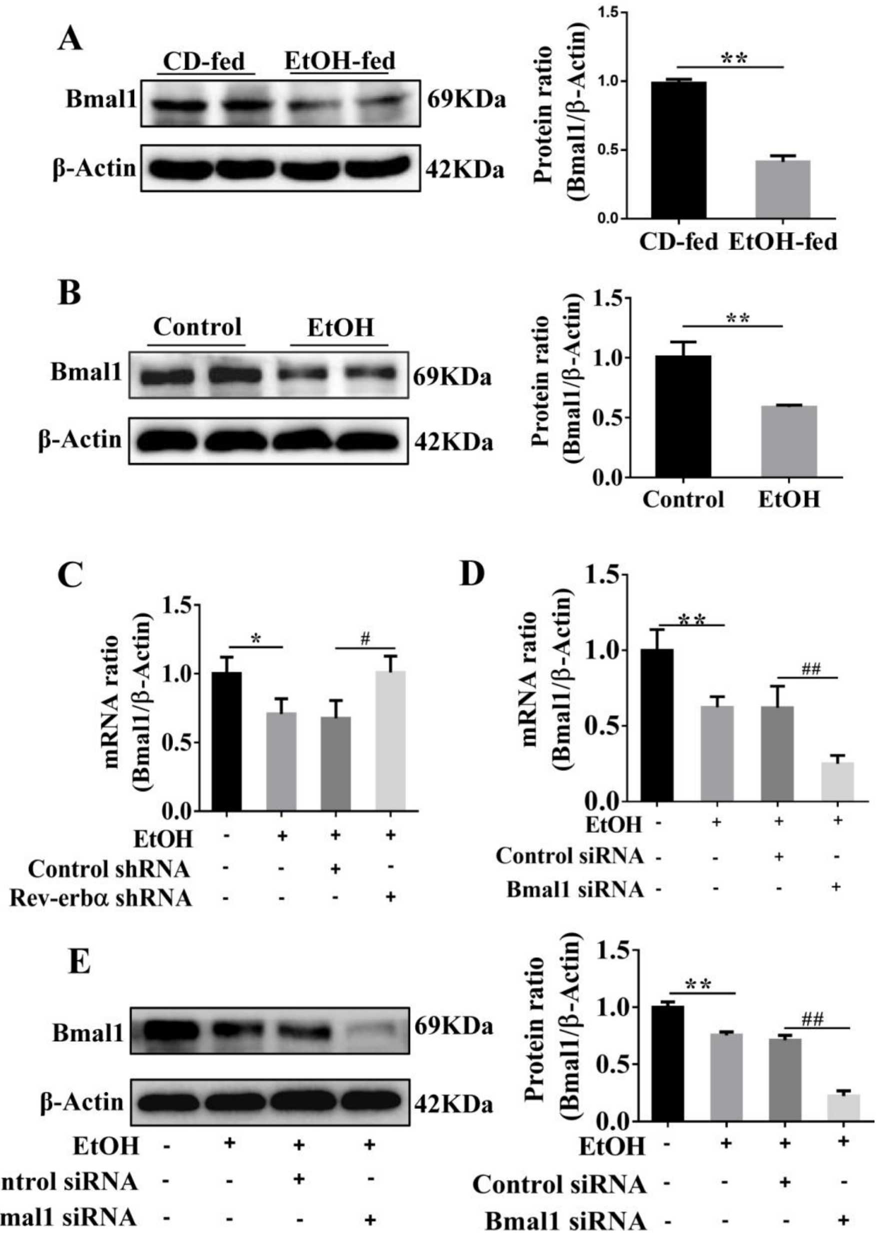

Figure S3. Bmal1 was up-regulated in EtOH-treated L-02 cells and was regulated by Rev-erbα. (A) Western blot analysis of Bmal1 in the liver of EtOH-fed mice ($n \geq 6$). (B) Western blot analysis of Bmal1 in EtOH-treated L-02 cells ($n \geq 3$). Bar represents the mean ± S.E.M.. Significance * $P < 0.05$, ** $P < 0.01$ vs. EtOH-fed group or EtOH group. (C) qRT-PCR analysis of Bmal1 in EtOH-treated L-02 cells transfected with Rev-erbα shRNA ($n \geq 3$). Bar represents the mean ± S.E.M.. Significance * $P < 0.05$, ** $P < 0.01$ vs. Control group, $^{\#}$ $P < 0.05$, $^{\#\#}$ $P < 0.01$ vs. Control shRNA group. (D) (E) qRT-PCR and western blot analysis of Bmal1 in EtOH-treated L-02 cells transfected with Bmal1 siRNA ($n \geq 3$). Bar represents the mean ± S.E.M.. Significance * $P < 0.05$, ** $P < 0.01$ vs. Control group, $^{\#}$ $P < 0.05$, $^{\#\#}$ $P < 0.01$ vs. Control siRNA group.
